# Modeling RNA polymerase interaction in mitochondria of chordates

**DOI:** 10.1186/1745-6150-7-26

**Published:** 2012-08-09

**Authors:** Vassily A Lyubetsky, Oleg A Zverkov, Sergey A Pirogov, Lev I Rubanov, Alexandr V Seliverstov

**Affiliations:** 1 Institute for Information Transmission Problems of the Russian Academy of Sciences (Kharkevich Institute), 19 Bolshoy Karetny per, Moscow, 127994, Russia

**Keywords:** RNA polymerase interaction, RNA polymerase competition, Transcription, Circular DNA, mtDNA in chordates, MELAS syndrome, Impact of DNA methylation, Hyposecretion of hormones, RNA interaction model, Polysome and ribonuclease interaction model

## Abstract

**Background:**

In previous work, we introduced a concept, a mathematical model and its computer realization that describe the interaction between bacterial and phage type RNA polymerases, protein factors, DNA and RNA secondary structures during transcription, including transcription initiation and termination. The model accurately reproduces changes of gene transcription level observed in polymerase sigma-subunit knockout and heat shock experiments in plant plastids. The corresponding computer program and a user guide are available at http://lab6.iitp.ru/en/rivals. Here we apply the model to the analysis of transcription and (partially) translation processes in the mitochondria of frog, rat and human. Notably, mitochondria possess only phage-type polymerases. We consider the entire mitochondrial genome so that our model allows RNA polymerases to complete more than one circle on the DNA strand.

**Results:**

Our model of RNA polymerase interaction during transcription initiation and elongation accurately reproduces experimental data obtained for plastids. Moreover, it also reproduces evidence on bulk RNA concentrations and RNA half-lives in the mitochondria of frog, human with or without the MELAS mutation, and rat with normal (euthyroid) or hyposecretion of thyroid hormone (hypothyroid). The transcription characteristics predicted by the model include: (i) the fraction of polymerases terminating at a protein-dependent terminator in both directions (the terminator polarization), (ii) the binding intensities of the regulatory protein factor (mTERF) with the termination site and, (iii) the transcription initiation intensities (initiation frequencies) of all promoters in all five conditions (frog, healthy human, human with MELAS syndrome, healthy rat, and hypothyroid rat with aberrant mtDNA methylation). Using the model, absolute levels of all gene transcription can be inferred from an arbitrary array of the three transcription characteristics, whereas, for selected genes only relative RNA concentrations have been experimentally determined. Conversely, these characteristics and absolute transcription levels can be obtained using relative RNA concentrations and RNA half-lives known from various experimental studies. In this case, the “inverse problem” is solved with multi-objective optimization.

**Conclusions:**

In this study, we demonstrate that our model accurately reproduces all relevant experimental data available for plant plastids, as well as the mitochondria of chordates. Using experimental data, the model is applied to estimate binding intensities of phage-type RNA polymerases to their promoters as well as predicting terminator characteristics, including polarization. In addition, one can predict characteristics of phage-type RNA polymerases and the transcription process that are difficult to measure directly, e.g., the association between the promoter’s nucleotide composition and the intensity of polymerase binding. To illustrate the application of our model in functional predictions, we propose a possible mechanism for MELAS syndrome development in human involving a decrease of Phe-tRNA, Val-tRNA and rRNA concentrations in the cell. In addition, we describe how changes in methylation patterns of the mTERF binding site and three promoters in hypothyroid rat correlate with changes in intensities of the mTERF binding and transcription initiations. Finally, we introduce an auxiliary model to describe the interaction between polysomal mRNA and ribonucleases.

## Background

In this work, we use the model developed and applied to study plant plastids [1] in the analysis of transcription in the mitochondria of chordates. The model is generally applicable to plastids and mitochondria, prokaryotes, and nuclear DNA. We also propose an auxiliary model describing a specific component of translation.

Many eukaryotes possess mitochondria, semi-autonomous organelles with a highly reduced genome. Animal mitochondria encode 22 tRNAs, 2 rRNAs and 13 proteins in a circular chromosome of 15–18 kbp. Transcription is conducted by phage-type RNA polymerases homologous to the polymerases of phages T7 and T3. Transcription initiation requires auxiliary protein factors and occurs at up to five distinct promoters, producing transcripts that can potentially be longer than the chromosome itself.

Among transcription factors are the mtTFA and mtTFB proteins, which bind the polymerase at each promoter [2,3]. These factors detach from the polymerase after transcription of 13 nucleotides. Several mtTFA isoforms are known that play a role in alternative splicing [4]. Two mtTFB isoforms, mtTFB1 and mtTFB2, are both involved in transcription initiation. However, the role of these transcription factors is not considered in this study.

Another essential initiation factor is mTERF [5], which also mediates transcription termination in the form of cooperative binding. This factor is an important component of our model. The properties of phage-type RNA polymerases have previously been studied [6-9]. In particular, in a head-on collision, two RNA polymerases approaching one another on the same DNA may pass by one another [6]. We assume that in this case the antisense mRNA forms a duplex and becomes inaccessible for the translation machinery.

**Figure 1:**
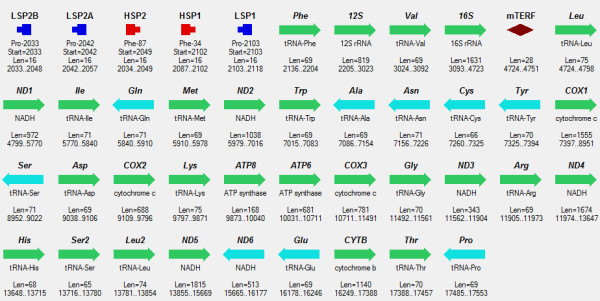
**Mitochondrial genome of *Xenopus laevis. ***The entire circular DNA is presented sequentially as four rows. Genes in the heavy strand (H-strand) are shown by green arrows, and genes in the light strand (L-strand) – by blue arrows. HSP1, HSP2 are two promoters in the H-strand. LSP1, LSP2A, LSP2B are three promoters in the L-strand. mTERF symbolizes a binding site of the protein factor mTERF acting as transcription terminator.

**Figure 2:**
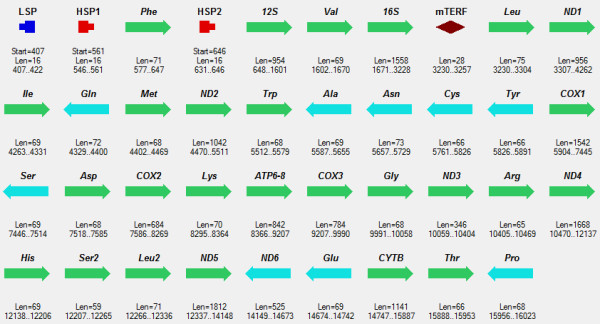
**Mitochondrial genome of *Homo sapiens. ***All symbols are the same as in Figure 1.

**Figure 3:**
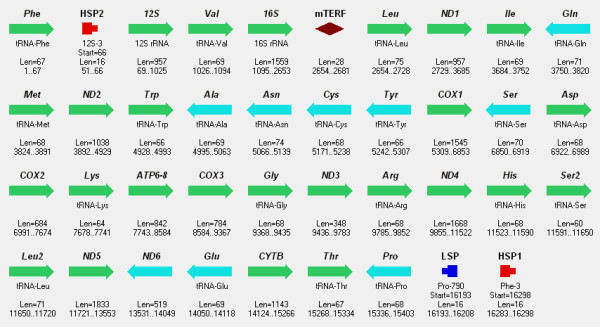
**Mitochondrial genome of *Rattus norvegicus. ***All symbols are the same as in Figure 1.

Our study focuses on mitochondria of human [*Homo sapiens*, Genbank:NC_012920.1], rat [ *Rattus norvegicus*, Genbank:NC_001665.2], and clawed frog [ *Xenopus laevis*, Genbank:NC_001573.1]. Data on the mitochondrial genome of mouse [ *Mus musculus*, Genbank: NC_005089.1] was also used as having the same gene order. These model organisms were chosen due to the availability of ample experimental evidence on RNA concentrations and half-lives that can be used to estimate gene transcription levels (transcription frequencies). The mitochondrial genomes of human, rat and frog are given in Figure 1, Figure 2, Figure 3, respectively. The RNA half-lives are described in Additional file 1 (Section 1).

### Structure and arrangement of mitochondrial promoters

Mitochondrial promoter locations differ substantially among species. Table 1 shows experimentally determined locations of mitochondrial promoters in human, rat, and frog. Human mitochondria possess three promoters, HSP1, HSP2, and LSP. Both the HSP1 and LSP promoters contain the conserved box 5′-CANACC(G)CC(A)AAAGAYA-3′ [10]. The transcription initiation site is located inside the conserved box 6–8 bases before 3'-end. The transcription initiation site is located at position 561 in HSP1 (upstream from gene tRNA-Phe), at position 646 in HSP2 (upstream from gene 12 S rRNA) [11], and at position 407 in LSP [12]. The promoter quality is affected by the regions: -16 – +7 for HSP1, and −28 – +16 for LSP [13].

**Table 1:** Transcription initiation sites in mitochondria

**Species**	**Sequence**	**Site**	**Position**
*Homo sapiens*	[Genbank:NC_012920.1]	HSP1	561
		HSP2	646
		LSP	(407)
*Rattus norvegicus*	[Genbank:NC_001665.2]	HSP1	16298
		HSP2	66
		LSP	(16193)
*Xenopus laevis*	[Genbank:NC_001573.1]	HSP1	2102
		HSP2	2049
		LSP1	(2103)
		LSP2A	(2042)
		LSP2B	(2033)

Positions in the complementary L-strand are shown in parentheses.

Rat mitochondria also possess three promoters [14]. The transcription initiation site is located at position 16298 in HSP1 (15 bases upstream from gene tRNA-Phe), at position 66 in HSP2 (upstream from gene 12 S rRNA), and at position 16193 in LSP.

Five promoters are known in frog mitochondria, HSP1, HSP2, LSP1, LSP2A, and LSP2B, all lying upstream of the tRNA-Phe gene [15,16]. Transcription is initiated at the conserved box ACRTTATA. Relative transcription initiation intensities have been estimated for the wild type and several mutant frog genotypes [17] (Table 2), and are illustrated in our Table 2 for the reader’s convenience.

**Table 2:** Transcription initiation intensities relative to the LSP1 promoter in frog mitochondria

**Promoter**	**Intensity (%)**
HSP1	13.6
HSP2	60.0
LSP1	100.0
LSP2A	16.6
LSP2B	38.2

Data are from [17].

### Effect of protein factors and mtDNA methylation on gene transcription levels

In early embryogenesis of frog a continuous increase of mtTFA concentrations is observed [18] associated with the elevation of gene transcription levels [19]. In the beginning of this period replication in mitochondria are almost arrested, and the initial pool of mitochondria is distributed between the dividing cells.

Hormone concentration, patterns of mtDNA methylation [14,20] and mutations substantially affect gene transcription.

Neither changes in mitochondrial gene expression levels during development in human [21,22], nor mitochondrial gene expression levels in different tissues of rat under different conditions [23,24] are currently included in our model.

### mTERF-dependent transcription termination

In human mitochondria, two terminators exist, each possessing different termination mechanisms. In the first mechanism, the mTERF protein binds to a 28 bp region immediately downstream of the 16 S rRNA gene; and located within the tRNA-Leu gene. This terminator is considered to be polarized and blocks almost all RNA polymerases on the DNA light strand but allows some to move through onto the heavy strand. A negligible portion of polymerases still passes through the terminator to transcribe the 16 S rRNA gene in the antisense direction [19]. The second mechanism, the mTERF-independent transcription termination due to G-quadruplex, is described in Additional file 1 (Section 2).

In mammals, there are two hypotheses about mechanism of transcription regulation on the heavy strand [5,14]. First, HSP1-initiated transcription may terminate after the rRNA gene, and longer transcripts are initiated at HSP2 only. Alternatively, all promoters may initiate longer transcripts, and some polymerases stop at mTERF regardless of the promoter.

Notably, in mammals mTERF binds cooperatively with the termination site and the mTERF activator site in the proximity of promoter HSP1, thus functioning as both terminator and activator [11].

The termination site is conserved and located downstream of the rRNA genes in many animals [25]. Similarly mTERF homologs have been detected in the nuclear genomes of many animals.

### MELAS syndrome

The A → G transition at position 3243, in the middle of the mTERF binding site, decreases mTERF’s affinity for DNA, thus causes a mitochondrial disorder. In human, this mutation causes: (i) a small decrease of rRNA transcription (12 S and 16 S, at similar levels), (ii) a decrease of tRNA-Leu concentration of up to 20%, (iii) a decrease in tRNA-Lys concentration of up to 50%, (iv) a slight decrease in total mRNA, and (v) a noticeable change in the amount of protein products [26]. These changes have to be reproduced by any model.

## Methods

### Input data, main model and its parameters

Complete mitochondrial genomes of human, rat, and frog analyzed in the study were obtained from GenBank, NCBI, [27], Figure 1, Figure 2, Figure 3.

Here, the protein factor under consideration is the multifunctional regulatory protein mTERF. G-quadruplex implicates DNA regions, but cross-hairpins typical for plastid [1] and bacterial genome DNA [28] are likely absent from the mitochondrial genomes of the animals under study.

Let us recall our model of interaction between RNA polymerases themselves, factors, and structures in DNA or RNA [1]. RNA polymerases can potentially attempt to bind with all promoters at a given locus. Each promoter is characterized by real number λ, the intensity of attempts to bind one of the surrounding polymerases. Formally, λ is the parameter of a Poisson stochastic process. An attempt is “successful” if the promoter is not already occupied by a polymerase or any other factor, even partially. After binding, polymerases move along the DNA strand and terminate at collisions with oncoming polymerases. They can also terminate at encountering protein factors or secondary structures. The abort process is not considered, as only phage-type RNA polymerases are present in mitochondria.

Similarly, protein factors attempt to bind their target sites. An attempt is “successful” if the site is not occupied by a polymerase or another protein. If a polymerase encounters a site bound with a protein factor, it either passes through and the protein-DNA complex dissociates, or it terminates and the complex survives. The analogous situation for cross-hairpins [1], is beyond the scope of the current study. Time intervals between any successive events in the model were estimated from distributions of stochastic and deterministic (polymerase movement) processes described above and were then summed up. Thus, each event can be described with a *modeled real time* commencing with the start of all modeled processes. The modeled real time of course does not coincide with the computation time, but it allows the transcription level (transcription frequency) for each gene to be computed in terms of the organism’s time.

The model has four *fixed parameters*: (i) the elongation rate (which is constant among chordates), (ii) the size of the phage-type RNA-polymerase, and (iii) the ratio of polymerases terminating at a G-quadruplex, (iv) the geometry of mtDNA, i.e., the location of genes, promoters and terminators. These parameters are discussed in the next section.

*Unknown parameters* are the transcription initiation (polymerase binding) attempt intensities at each promoter, mTERF protein terminator binding attempt intensities, and the conditional (provided that mTERF is already bound) probabilities *p* and *q* of passing the mTERF-dependent terminator in both directions. The model contains no other parameters. All gene transcription levels are estimated in the model using the values of these parameters.

The model software allows the user to specify physical time of modeling along a trajectory, the model run-up time, the number of trajectories to average, etc. The main executing parameters are the number of processors and time to halt modeling and create a checkpoint.

The *solution* to the model is a set of parameters, including intensities of binding attempts to all existing promoters, conditional probabilities *p* and *q* of passing the mTERF-dependent terminator in both directions, and intensity *λ* of mTERF binding attempts. Here *λ* incorporates the process of spontaneous dissociation of the mTERF·DNA complex. Table 3 shows conditional probabilities *p* and *q* ( *passage probabilities*) inferred under two RNA polymerase movement rates.

**Table 3:** The mTERF terminator passage probabilities on the heavy and light strands

**Rate, nt/s**	***p***	***q***	***L***_**1**_ ***n*** **(Frog1)**	***L***_**1**_ ***n*** **(Frog2)**	***L***_**1**_ ***n*** **(Frog3)**	***L***_**1**_ ***n*** **(total)**
500	0.0164	0.0056	11.243	3.193	0.043	2.098
200	0.2165	0.0015	10.844	3.240	0.309	2.235

The minimum of *L*_1_*n* for three frog specimens, and minimum of *L*_1_*n*(total) are shown. For comparison, the same minima are given for the RNA polymerase elongation rate of 200 nt/s.

The modeling procedure details are described in Additional file 1 (Section 3).

Comparing the model and experiment requires knowledge of RNA half-lives. This was not considered in the original model description [1]. Notably, for many genes, the transcript concentration relative to the null time point or to a reference RNA concentration (*relative RNAconcentration*), and sometimes the RNA half-life is known in a stationary state.

This allows a gene’s transcription level to be correlated with its RNA concentration and half-life.

In frog, the RNA concentration *u*_*ij*_ is known for the *j*-th gene at the *i*-th time point relative to its RNA concentration at null time point, i.e., $u_{\mathrm{ij}}=\frac{2Z_{\mathrm{ij}}\cdot t_{j}}{2Z_{\mathrm{oj}}\cdot t_{j}}=\frac{z_{\mathrm{ij}}}{z_{\mathrm{oj}}}$, where *z*_*ij*_ is the transcription level of the *j*-th gene at the *i*-th time point, *t*_*j*_ is the RNA half-life of the *j*-th gene. Absolute half-lives *t*_*j*_ are unknown. Table 4 provides experimental ratios *u*_*ij*_ (errors not estimated) and ratios $\frac{z_{\mathrm{ij}}}{z_{\mathrm{oj}}}$ of mean values *z*_*ij*_, and they are compared to each other. The values *z*_*ij*_ themselves are in Additional file 2.

**Table 4:** Agreement between the model and experiment for three frog specimens

**time**	***mTERF***	***LSP1***	**ND1**			**COX2**			**ATP6/8**			**ND4**			**ND6**			**CYTB**		
	**Frog1**		**mod**	**exp**	**dev**	**mod**	**exp**	**dev**	**mod**	**exp**	**dev**	**mod**	**exp**	**dev**	**mod**	**exp**	**dev**	**mod**	**exp**	**dev**
Egg	**0.0157**	**0.0034**	1.0	1.0		1.0	1.0		1.0	1.0		1.0	1.0		1.0	1.0		1.0	1.0	
+5 h	**0.0448**	**0.0089**	1.0	1.1	−12	0.9	0.8	+14	0.9	0.9	+1	0.9	2.1	−59	2.4	2.4	−1	0.8	0.7	+19
+10 h	**0.0872**	**0.0157**	1.2	1.3	−5	1.1	1.1	+1	1.1	0.7	+56	1.0	2.3	−57	4.1	4.0	+2	0.9	0.6	+50
+14 h	**0.0793**	**0.0173**	1.7	2.3	−26	1.6	1.6	−3	1.5	1.3	+18	1.4	3.0	−53	4.4	4.4	0	1.2	1.2	+3
+16 h	**0.0960**	**0.0209**	2.0	2.9	−31	1.7	1.4	+24	1.7	1.3	+31	1.5	4.3	−65	5.6	5.8	−4	1.3	1.3	+2
+18 h	**0.0542**	**0.0157**	2.1	3.2	−34	1.9	1.7	+14	1.9	1.9	+1	1.8	4.5	−60	4.4	4.2	+4	1.6	1.3	+25
+20 h	**0.0655**	**0.0157**	1.8	3.0	−41	1.6	1.4	+13	1.6	1.8	−12	1.5	4.6	−68	4.2	4.2	0	1.3	1.2	+8
+23 h	**0.0721**	**0.0492**	9.4	9.7	−4	7.6	5.1	+49	7.4	6.5	+14	6.4	16.1	−60	12.9	12.2	+5	5.3	5.2	+2
+48 h	**0.0542**	**0.0872**	29.3	26.6	+10	26.2	13.4	+96	26.0	26.1	0	23.8	60.3	−61	18.6	18.6	0	20.2	23.4	−14
+96 h	**0.0407**	**0.0960**	48.1	48.7	−1	45.3	20.9	+117	45.4	48.3	−6	43.3	104.2	−58	16.7	17.4	−4	38.8	39.3	−1
**time**	***mTERF***	***LSP1***	**ND1**			**COX2**			**ATP6/8**			**ND4**			**ND6**			**CYTB**		
	**Frog2**		**mod**	**exp**	**dev**	**mod**	**exp**	**dev**	**mod**	**exp**	**dev**	**mod**	**exp**	**dev**	**mod**	**exp**	**dev**	**mod**	**exp**	**dev**
Egg	**0.0089**	**0.0041**	1.0	1.0		1.0	1.0		1.0	1.0		1.0	1.0		1.0	1.0		1.0	1.0	
+6 h	**0.0045**	**0.0023**	1.2	1.3	−8	1.2	1.0	+22	1.2	1.3	−5	1.2	1.4	−12	0.7	0.7	+6	1.2	1.2	+3
+9 h	**0.0073**	**0.0045**	1.3	1.5	−14	1.3	1.3	−1	1.3	1.2	+8	1.3	1.6	−19	1.1	1.1	+1	1.3	1.3	−1
+20 h	**0.0157**	**0.0157**	3.8	4.6	−17	3.7	3.7	+1	3.7	3.7	+1	3.7	3.7	0	2.8	2.8	−2	3.6	4.0	−11
+30 h	**0.0157**	**0.0230**	7.2	7.2	0	7.1	6.8	+4	7.1	8.1	−13	7.0	6.2	+14	3.7	3.7	0	6.8	8.1	−17
+48 h	**0.0407**	**0.1056**	20.5	19.5	+5	19.7	19.7	0	19.6	28.7	−32	19.1	17.7	+8	8.6	8.4	+2	17.3	23.1	−25
+7 days	**0.0041**	**0.0073**	6.5	6.1	+7	6.6	8.0	−18	6.6	8.5	−22	6.7	4.9	+36	2.4	2.3	+3	6.6	6.6	+1
**time**	***mTERF***	***LSP1***	**16 S**			**ND6**														
	**Frog3**		**mod**	**exp**	**dev**	**mod**	**exp**	**dev**												
Egg	**0.0960**	**0.0026**	1.0	1.0		1.0	1.0													
+5 h	**0.0407**	**0.0050**	2.2	2.2	+0.9	2.2	2.2	0.0												
+14 h	**0.0230**	**0.0081**	5.0	5.0	0.0	4.5	4.5	−0.2												
+20 h	**0.0038**	**0.0028**	5.9	6.0	−1.3	4.0	4.0	+0.5												
+28 h	**0.0336**	**0.1056**	92.2	92.0	+0.2	25.1	25.0	+0.4												
+48 h	**0.0143**	**0.0306**	44.1	44.0	+0.2	15.0	15.0	+0.3												

In columns: “time” gives absolute times of embryonic development; “*mTERF*“ and “ *LSP1*” are intensities of the mTERF factor binding with the termination site and cooperative binding with the promoter, respectively, under the polymerase rate of 500 nt/s; further columns are modeled (mod) and experimental (exp) gene transcription levels and their relative deviations (dev) obtained with equation (9). Variables are in bold.

In human, RNA concentration *u*_*j*_ relative to the reference gene ND1 and RNA half-lives are known in a stationary state, i.e., $u_{j}=\frac{2z_{j}\cdot t_{j}}{2z_{0}\cdot t_{0}}$, where *z*_*j*_ is the transcription level of the *j*-th gene, and *t*_*j*_ is the RNA half-life of the *j*-th gene. The ratio $\frac{z_{j}}{z_{0}}$ is estimated in the model and compared with the experimentally known

(1) $$u_{j}\cdot \frac{t_{0}}{t_{j}}\text{,}$$

in Table 5, upper rows. The special case of gene COX1 is discussed in Additional file 1 (Section 3).

**Table 5:** Agreement between the model and experiment for healthy human and human with MELAS syndrome

**Solution parameters for healthy human**						**Transcription levels (relative to ND1 gene) in the model (1st row) and experiment (2nd row)**						
***LSP***	***HSP1***	***HSP2***	***mTERF***	***R***	***L***_**1**_ ***n***	**ND2**	**COX1**	**COX2**	**ATP6/8**	**ND3**	**ND5**	**CYTB**
**0.0031**	**0.0031**	**0.0126**	**0.6456**	23.955	1.945	1.00	1.00	1.00	0.96	0.96	0.96	0.96
Experimental estimates	Transcription level:					1.40	1.04	1.72	0.91	1.04	1.86	2.31
	Error (if statistically independent):					±0.23 (1.8)	±0.52 (0.1)	±0.61 (1.2)	±0.43 (0.1)	±0.12 (0.7)	±0.56 (1.6)	±0.56 (2.4)
	Error (if statistically dependent):					±0.40 (1.0)	±0.82 (0.1)	±0.95 (0.8)	±0.71 (0.1)	±0.20 (0.4)	±0.99 (0.9)	±1.01 (1.3)
Deviation from the experiment,%:						−29	−4	−42	+5	−4	−48	−58
**Solution parameters for MELAS case**						**Changes of transcription levels in the model**						
						**Phe**	**12 S**	**Val**	**16 S**	**Leu**	**Lys**	**CYTB**
**0.0031**	**0.0004**	**0.0126**	**0.5336**	24.333		3.84	1.20	1.20	1.20	1.16	1.22	1.17

Inferences are obtained under the polymerase rate of 500 nt/s and the values of *p*, *q* estimated for the frog.

**Left panel**: the solutions for healthy human (upper part) and human with MELAS syndrome (lower part). Variables and predicted values are in bold. Intensities decrease 7.75 times for *HSP1* and 1.21 times for *mTERF*; RNA/RNA_−_ =1.18, *R =* 24. The *L*_1_*n* minima differ by 2.4% if general conditions are only imposed.

**Right panel (upper part)**: Transcription levels of seven genes (relative to ND1 gene) in the model and experiment for healthy human. Experimental errors were estimated for both cases of statistically independent measurements and counter assumption. In the latter case, the model results are within experimental error, except for the CYTB gene. Differences between model and experimental estimates are shown in parentheses in the error radius units.

**Right panel (lower part)**: For selected genes, decrease of their transcription levels in the model in MELAS case comparing to healthy human. The transcription level of tRNA-Phe drops 3.8-fold, of 12 S and 16 S – 1.2-fold. The Leu and Lys transcription levels decrease 1.2-fold, which agree with experimental observations.

In both rats, mRNA concentrations of the genes COX1, ATP6/8, COX3, ND4, ND5, CYTB were measured individually relative to 16 S rRNA. Each ratio in the hypothyroid rat was calculated as percentage of the corresponding ratio in the euthyroid rat, Table 6. Thus, the experimentally measured ratio is $u_{j}=\frac{\left({z_{j}}^{h}{t_{j}}^{h}\right)\left({z_{0}}^{e}{t_{0}}^{e}\right)}{\left({z_{0}}^{h}{t_{0}}^{h}\right)\left({z_{j}}^{e}{t_{j}}^{e}\right)}$, where *z*_*j*_ is the transcription level of the *j*-th gene in hypothyroid ( *h*) or euthyroid ( *e*) rats, *j* = 1,…,6; *z*_*0*_ is the 16 S rRNA transcription level; *t* are half-lives specific to *j*, *e*, *h*. Therefore, the value $\frac{z_{j}^{h}z_{0}^{e}}{z_{0}^{h}z_{j}^{e}}$ estimated in the model was compared with the experimentally obtained

(2) $$u_{j}\cdot \frac{t_{0}^{h}t_{j}^{e}}{t_{j}^{h}t_{0}^{e}}$$

**Table 6:** Experimental data on mitochondrial transcripts in rat

**Gene**	**Euthyroid**		**Hypothyroid**	
	**Normalized mRNA/rRNA ratio**	**Half-life (min)**	**Normalized mRNA/rRNA ratio**	**Half-life (min)**
16 S		44.48 ± 6.34		87.50 ± 27.52
COX1	100 ± 16	84.41 ± 27.49	86 ± 13	235.12 ± 48.68
ATP6/8	100 ± 19	78.14 ± 21.05	59 ± 9	277.52 ± 31.58
COX3	100 ± 19	78.14 ± 21.05	59 ± 9	277.52 ± 31.58
ND4/4 L	100 ± 16	84.41 ± 27.49	86 ± 13	235.12 ± 48.68
ND5	100 ± 25	46.00 ± 10.41	52 ± 11	60.52 ± 5.92
CYTB	100 ± 27	63.70 ± 7.82	57 ± 7	204.30 ± 28.64

Data are from [14]; the ± signs stand for standard deviations. For each gene, mRNA/rRNA ratios were normalized with euthyroid rat values taken for 100%.

The numerator and denominator of the ratio are both unknown.

Note that, when comparing gene transcription levels in model and experiment, the choice of functional is not straightforward. Consider the natural functional

(3) $$L_{1}n=\sum_{ji}\frac{\left|x_{\mathrm{ji}}-y_{\mathrm{ji}}\right|}{\max\left\{x_{\mathrm{ji}},y_{\mathrm{ji}}\right\}}\text{,}$$

Where *x*_*ji*_ and *y*_*ji*_ are relative transcription levels in gene *j* at time *i* (if no time data, the *i* index is omitted) in the model and the experiment. To compare data on three experimental frogs simultaneously, metric (3) can be generalized:

(4) $$L_{1}n\left(total\right)=\sum_{k=1}^{3}\left(\frac{1}{n_{k}\cdot s}\sum_{ji}\frac{\left|x_{\mathrm{ji}}-y_{\mathrm{ji}}\right|}{\max\left\{x_{\mathrm{ji}},y_{\mathrm{ji}}\right\}}\right)$$

where *n*_*k*_ is the “dimension” of compared data, $s=\sum_{k=1}^{3}\frac{1}{n_{k}}$. The dimensions are, n_1_ = 54 (nine time points, six genes), n_2_ = 36 (six time points, six genes), n_3_ = 10 (five time points, two genes), respectively.

We also considered some other functionals, listed in Additional file 1 (Section 4), which produced similar solutions. Hereafter, only functionals (3)–(4) are discussed.

### Fixed parameters of the main model

All model parameters are listed in the previous section.

As for the mtDNA geometry, sequence data was obtained from GenBank, NCBI, [27]. Multiple alignments were constructed with MEGA 5 [29], in order to detect terminator sequences.

The ratio of polymerases terminating at a G-quadruplex is discussed in Additional file 1 (Section 1).

To be formal, we should include the RNA polymerase elongation rate in unknown parameters, and the rate should also be varied. Because of large computations, in current study we examined only two values of the rate, 200 nt/s and 500 nt/s. The rate is unlikely to be less than 200 nt/s, but values greater than 500 nt/s are possible (see the next paragraph). A preliminary prediction of the model is that the polymerase elongation rate is 500–800 nt/s.

#### The elongation rate of phage type RNA polymerase (NEP)

The NEP rate is a key parameter in our model, but no experimental data of this rate is available to the best of our knowledge. The rate of replication fork progression in *E. coli* is 1500 nt/s, which is assumed to be the maximal NEP rate. A lower bound for this rate can be estimated from the ratio of length *E* of the first exon and length *I* of the first intron in protein-coding genes in plastids of the *Streptophyta* lineage. In plastids, transcription and translation processes are concurrent, and transcription of the first intron must end before translation of the first exon starts. Therefore, in the absence of translational regulation, the ratio of the polymerase and ribosome movement rates must exceed ( *E* + *I*)/ *E*. Such regulation was predicted for genes *atpF*, *clpP*, *petB*, *petD*[30]. For genes with short first exons, a delay in translation initiation must occur. Therefore, to estimate the lower bound of NEP rate, only genes unlikely to possess such regulation or trans-splicing should be used (e.g. *rpoC1*, *infA*, *ndhA*, *ndhB* in *Arabidopsis thaliana*). NEP-transcription is experimentally shown for *rpoC1*[31] and can be suggested for other genes because of the lack of quality PEP promoters. In different *Streptophyta* species the maximal ratios are 1.08 ( *infA* in *Cucumis melo*), 3.71 ( *ndhA* in *Chara vulgaris*), 3.75 ( *rpoC1* in *Zygnema circumcarinatum*), 3.93 ( *ndhB* in *Olea europaea*). The greatest of these ratios, 3.93, corresponds to the lowest NEP rate of 177 nt/s. Higher ratios are found in algae with uncharacterized transcriptional processes: 7.86 ( *rpl2* in *Chara vulgaris*), 7.94 ( *ycf3* in *Zygnema circumcarinatum*), 10.27 ( *ycf66* in *Zygnema circumcarinatum*). The greatest ratio suggests an NEP rate of 462 nt/s. Further knowledge of the NEP rate can improve the model accuracy.

Therefore, the lowest NEP rate of 500 nt/s and the control rate of 200 nt/s were assumed. The model implementation enables the NEP rate and other parameters to be varied.

#### The size of phage type RNA polymerase (NEP)

The NEP size is assumed equal to the size of the phage T7 polymerase. The promoter size was experimentally identified as −14 to +1 bp (relative to the transcription initiation site) in mutagenic studies of the phage T7 NEP ortholog in tobacco chloroplasts [7]. The −15 position indicates a slight effect on the promoter quality [7]. Footprinting studies suggest that 15 DNA bases are occupied by NEP or that 11 bases are unpaired [8]. The estimate of 15 bases was obtained in X-ray structure analysis of the phage T7 polymerase [9]. The current study assumes the NEP occupancy to be −15 to +1.

### Model of a polysome and ribonuclease interaction (auxiliary model)

To explain the MELAS phenotype, an auxiliary model was developed that describes the interaction between polysomal mRNA and ribonuclease.

The following expression describes time τ of any mRNA half-life, in terms of elementary probabilities (see Additional file 3):

(5) $$\tau =\frac{1}{\mu }\left(1+d\lambda \right)\exp\left(w\lambda \right)\ln2\text{,}$$

where $\lambda =\frac{\nu N}{1+\alpha N}$ is the intensity of ribosome binding to its site, ν is a specific intensity under low *N*, where *N* is the number of ribosomes in a healthy human mitochondrion, *α* is a Michaelis-Menten dependency parameter (saturation over *λ* occurs at high *N* and equals $\frac{\nu }{\alpha }$). Then, *w* is the ratio of the linear size *h* (in codons) of the ribonuclease on mRNA to the rate *V* of the ribosome elongation ( *V* = 15 codons per second, *h* = *Vw* = 15 *w*), *d* is the ratio of size *h*_1_ of the ribosome on mRNA to the ribosome elongation rate *V* ( *h*_1_ = 10 codons, *h*_1_ = *Vd*), *μ* is the interaction intensity of the ribonuclease with a specific mRNA site that leads to RNA cleavage. It is clear that *w* cannot be less than 1/15 s, and it is likely less than 4/3 s; *N* depends on the expression of other genes, especially ribosome genes, its value is inferred in the main model as absolute concentrations of 12 S or 16 S rRNA. Parameters *ν*, *α* and *μ* depend on the mRNA sequence. Unfortunately, values of *ν* and *α* are unknown. Note that *ν* and *α* depend on the ribosome binding site, i.e., in the general case those are *ν*_*j*_ and *α*_*j*_ over *j* RNAs. It is plausible that *μ* is the same for healthy and diseased humans.

Although here only the ribonuclease is considered as a factor in mRNA cleavage, other factors can be considered analogously.

Similarly, in diseased human, the mRNA half-life *τ*′ is expressed in terms of *N*′, the number of ribosomes in the mitochondrion, thus

(6) $$\frac{1+d\lambda }{1+d\lambda^{\prime }}\exp\left[w\left(\lambda -\lambda^{\prime }\right)\right]=\frac{\tau }{\tau^{\prime }}\text{.}$$

The left hand of (6) is evidently more than one, *τ*/ *τ*′ > 1. For at least one mRNA, the experimentally expected interval of *τ*/ *τ*′ is 1.5–3 [26]. For that mRNA, it follows from equation (6), that a small change in the absolute number *N* of ribosomes greatly influences the mRNA half-life, and thus the amount of the corresponding protein. The model solution confirms that the half-lives of rRNA and most mRNAs are similar in healthy and diseased humans. The disease phenotype might require a sharp decrease in half-life of a single (even short) mRNA (see item 6 in Additional file 1, Section 3). This suggests a plausible explanation for the MELAS phenotype.

The intensity of RNA decay by the ribonuclease is given by:

(7) $$\kappa =\frac{\mu }{1+d\lambda }\cdot \exp\left(-\lambda w\right)\text{.}$$

Each mRNA was assumed above to possess a single ribonuclease site. To the best of our knowledge the actual number and arrangement of cleavage sites on RNA remain to be determined. However, if the average number of cleavage sites per RNA for a given gene among all mitochondria in a tissue is *k*, then in the above equation *κ* is substituted by *κk*. This conclusion and equations (5) to (7) are derived in Additional file 3.

An RNA *window* is defined as a region of a minimum length *h* = *Vw*, that separates two neighboring ribosomes, where *Vw* is the linear size of the ribonuclease on mRNA.

By combining equations (5) and (6) for different RNAs, *ν*_*j*_ can be expressed in terms of *w*.

### Comparison to the experiment

The distributions of variables $u_{j},t_{0},t_{j}$ are not experimentally known. This precludes us from estimating confidence for experimental values (1) or (2) on the basis of probability-theoretical methods which are usually applied to compare predictions with experiments. However, *absolute errors* can be used instead. Let Δ be an absolute error of *b* (expressed as (1) or (2)) and *a* is a model value for the same expression, then it can be verified if:

(8) "abelongs withinb±Δ"

The error Δ of expression value (1) or (2) is then estimated trivially [32] by using one of two common empirical relations. First, the error of an algebraic sum is $\Delta \left(x\pm y\right)=\sqrt{\Delta {\left(x\right)}^{2}+}\Delta {\left(y\right)}^{2}$ if summand errors are statistically independent. Alternatively, $\Delta \left(x\pm y\right)\leq \Delta \left(x\right)+\Delta \left(y\right)$. The error of product x·y or ratio *x*/ *y* is $\Delta \left(x\circ y\right)=\left(x\circ y\right)\cdot \sqrt{{\left(\Delta \left(x\right)/x\right)}^{2}+{\left(\Delta \left(y\right)/y\right)}^{2}}$ if member errors are statistically independent. Otherwise, $\Delta \left(x\circ y\right)\leq \left(x\circ y\right)\cdot \left(\Delta \left(x\right)/x+\Delta \left(y\right)/y\right)$. Therefore, either a hypothesis of statistical independence is assumed to be true and equalities apply, or inequalities are used, in which case expression (8) becomes uncertain. Fortunately, both cases produce similar results.

The experimental and model values of (1) and (2) and their errors are given in Table 5 and Table 7, assuming that constituent errors are either statistically independent, or not. The model predictions fall within the error intervals b±1.3Δ and b±2.4Δ, respectively (Table 5 and Table 7).

**Table 7:** Agreement between the model and experiment for euthyroid and hypothyroid rats

***LSP***	***HSP***	***mTERF***	***R***	***L***_**1**_ ***n***	**Transcription levels in hypothyroid rat relative to euthyroid one in the model (upper) and experiment (lower)**					
					**COX1**	**ATP6/8**	**COX3**	**ND4**	**ND5**	**CYTB**
**0.1056**	**0.0721**	**0.9453**	30.605	1.736	0.666	0.641	0.646	0.622	0.614	0.613
**0.1056**	**0.0336**	**0.9453**	30.637		0.61	0.33	0.33	0.61	0.78	0.35
Error (if statistically independent):					±0.35 (0.2)	±0.17 (1.9)	±0.17 (1.9)	±0.34 (0.0)	±0.42 (0.6)	±0.17 (1.5)
Error (if statistically dependent):					±0.79 (0.1)	±0.39 (0.8)	±0.39 (0.8)	±0.79 (0.0)	±0.97 (0.2)	±0.39 (0.7)
Deviation from the experiment,%:					+9	+94	+96	+2	−21	+75

Inferences are obtained under the polymerase elongation rate of 500 nt/s and the values of *p*, *q* estimated for the frog.

**Upper left part**: the solutions for the euthyroid (upper row) and hypothyroid (lower row) rats. Variables and predicted values are in bold. Note that *HSP* = *HSP1* + *HSP2*.

**The right part**: experimental data and model predictions. Experimental errors were estimated for both cases of statistically independent measurements and counter assumption. In the latter case, the model results are within experimental error. Differences between model and experimental estimates are shown in parentheses in the error radius units.

Importantly, experimental and predicted solutions from Table 4, Table 5, Table 7 differ “insignificantly” in another respect. The percentage concordance between values *a* and *b* (empirical and predicted) can be expressed as:

(9) $$\left(a-b\right)\cdot 100/b\text{.}$$

The sign of this value indicates a decrease or increase of *a* relative to *b*. As a rule-of-thumb, changes in gene transcription level that are between −50% (i.e. halving) and +100% (i.e. doubling) are considered “insignificant” [33]. By this measure, almost all model predictions differ insignificantly from experimental data (Table 4, Table 5, Table 7).

### Model implementation

The model was implemented in C++ in two versions (command line interface and GUI) and is distributed freely under the GNU General Public License v.3 on the web page [34]. Similar to the earlier release [1], it is an event-driven automaton that simulates large combinations of interacting stochastic and deterministic processes in a DNA locus against modeled physical time. RNA polymerase binding is modeled as a stochastic process. The subsequent polymerase elongation in this study is modeled as a deterministic process with constant rate. The following collision events are modeled: (i) a polymerase or factor attempts to bind a previously occupied site, (ii) secondary structures attempt to form within a bound site, and (iii) two oncoming polymerases attempt to process the same nucleotide. Scenarios and rules of the collision’s resolution are customizable model options.

The events in the model are handled chronologically, and ordered in a complex system of partially ordered queues. The program performance depends heavily on the rate of serving the queues.

Polymerase interaction was previously studied within short loci of a few thousand base pairs [1]. Here we model transcription along complete mitochondrial genomes of up to 18 kbp in length. The circular arrangement of this DNA introduces a novel scenario: polymerases can continue transcription until they complete several circles and collide, which considerably increases the number of simultaneously modeled events.

Another important aspect of our model is that we considered phage-type RNA polymerase. Although its elongation rate is not experimentally known it is likely higher than that of the bacterial-type polymerase. In plastids, transcription is carried out by polymerases of both types. However, a faster polymerase does not make a difference, as it cannot outpace or influence the slower polymerase. In this study, elongation rates of 200 nt/s and greater were assumed, which is an order of magnitude higher than in the earlier modeling attempts. This led to a higher rate of access to larger event queues and therefore to reduced performance. Queue processing was considerably improved in the current implementation by changing from a linear event queue to a system of partially ordered queues, which allowed us to attain previously observed levels of performance [1].

In our previous implementation, any polymerase collision with a factor or secondary structure terminated transcription [1]. The current model implements a new class of objects, protein terminators with nonzero passage probabilities in both directions; these probabilities *p* and *q* are the terminator parameters mentioned under Methods.

The model constructs an individual trajectory in the event space and estimates gene transcription levels along the trajectory. Under the same set of model parameters, the levels are averaged over multiple trajectories. Computations can be effectively parallelized on a cluster with MPI v. 1.2 or newer. In this study the results were obtained on 2048 CPUs at the MVS-100 K supercomputer of the Joint Supercomputer Center of the RAS [35].

The inverse problem was solved by multi-objective optimization. For example, the response landscape for functional (4) is complex, with numerous valleys and local minima, which excludes the use of standard (e.g. gradient-based) local minimization techniques and requires heuristic solutions. Our approach is based on the following effective strategy: promoters in both strands of mtDNA are concentrated in a region containing only Phe-tRNA genes in human and rat, which produces opposite flows of polymerases that compete mostly outside this region. Therefore, a lack of transcription on one strand indicates that it is blocked by a heavy flow of polymerases along the opposite strand. Under the general assumption of non-zero transcription level, further increase of promoter binding intensities on the successful strand is not reasonable and does not lead to a better solution. This fact considerably reduces computational complexity. For any current set of model parameters, promoter binding intensities are varied in each direction only until transcription stops for one of the genes. This optimization strategy is referred to as “active search”.

## Results

Given an RNA polymerase transcription rate of 500 nt/s, one solution was obtained for each model organism, the frog (three embryos), human (healthy and diseased), and rat (eu- and hypothyroid).

In all cases the mTERF terminator passage probabilities (i.e., the portion of RNA polymerase passages through bound mTERF) were *p* = 0.0164 on the heavy strand and *q* = 0.0056 on the light strand, indicating a triple-fold polarization of the terminator.

**Figure 4:**
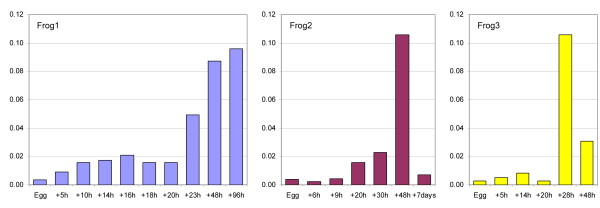
**LSP1 promoter binding intensities plotted against time for three frog embryos.** The LSP1 promoter binding intensities mostly increase in agreement with [19].

In frogs the LSP1 promoter binding intensities mostly increase with time (Table 4 and Figure 4). Predicted and experimental transcription levels are in good agreement, with the exception of the estimate obtained with equation (9) for the first frog. In this case, at 96 hours of development the difference slightly exceeds +100%; for gene ND4 at all time points the difference slightly exceeds −50%, Table 4.

In healthy human the *LSP*, *HSP1*, *HSP2* and *mTERF* binding intensities are 0.0031, 0.0031, 0.0126, and 0.6456, respectively (Table 5). In the case of MELAS syndrome the *HSP1* and *mTERF* intensities drop 7.75- and 1.21-fold and become 0.0004 and 0.5336, respectively. The *ratio R* of gene 12 S to gene COX2 transcription levels is 24, and the *ratio* RNA/RNA_−_ of weighted total RNA concentrations in healthy and diseased human is 1.18 (these denotations, *R* and RNA/RNA_–,_ are explained and justified in Additional file 1, Section 3). Transcription levels of tRNA-Phe and 16 S rRNA dropped in diseased human 3.84- and 1.2-fold, respectively. The decrease in tRNA-Leu and tRNA-Lys transcription was 1.2-fold, which is within experimental error. The functional value for optimal solution under all imposed *additional conditions* vs. only *general conditions* (ref. definitions in Additional file 1, Section 3) differs by 2.4%.

All predicted and experimental transcription levels in healthy human are within experimental error, except for *CYTB*, for which this error is exceeded by 29%. In addition *CYTB* exhibits a difference of around −50% in healthy human as estimated with equation (9), Table 5. We will revert to the case of *CYTB* in the Discussion.

In rats, we defined *HSP* as the total intensity of binding attempts to promoters HSP1 and HSP2, i.e. *HSP = HSP1* + *HSP2*. In euthyroid rat, the binding intensities for *LSP*, *HSP* and *mTERF* are 0.1056, 0.0721 and 0.9453, respectively. In hypothyroid rat, *HSP* drops to 0.0336 (Table 7). The ratio *R* of 12 S and COX2 transcription levels is 30.605 in euthyroid rat and slightly increases to 30.637 in hypothyroid one. Similarily, differences in predicted and experimental transcription levels between the eu- and hypothyroid animals are within experimental error. Differences obtained with equation (9) are insignificant, Table 7.

**Figure 5:**
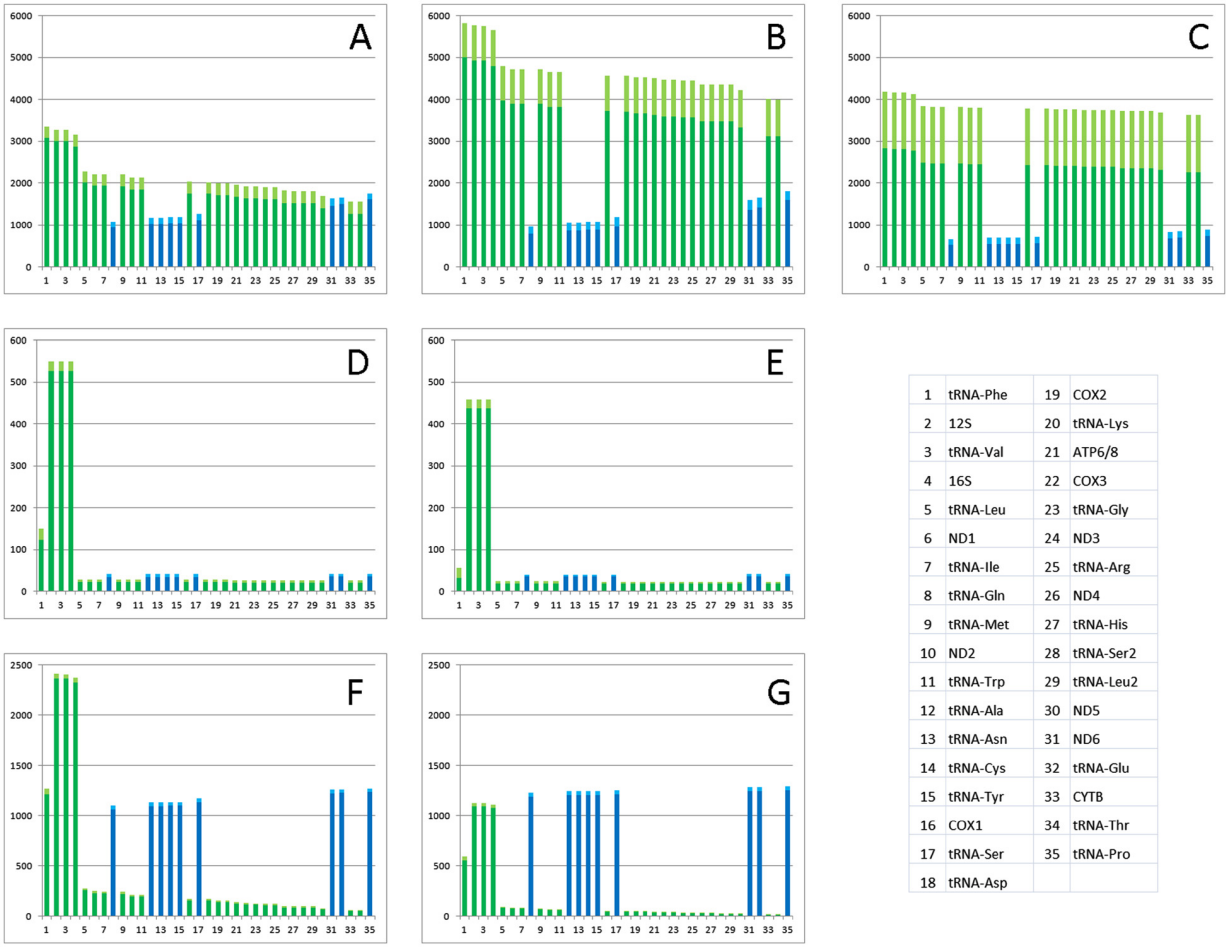
**Transcription numbers predicted during 9 hours of modeled physical time.A**, **B**, **C** – frog embryos 1, 2, 3; **D** – healthy human; **E** – human with the MELAS phenotype; **F** – euthyroid rat; **G** – hypothyroid rat. Genes on the heavy strand are in green, on the light strand – in blue. Shaded are mean values, not shaded – standard errors.

The model’s solutions and comparisons to experimental values are presented in Table 3, Table 4, Table 5, Table 7. The predicted absolute transcription levels of all genes are illustrated in Figure 5 and detailed in Additional file 2. Importantly, most predictions fall within experimental error. This definition, however, needs further justification. Specifically, a more accurate error estimation would require knowledge of the distribution of the experimental measurements (see Section 4 of the Methods).

## Discussion

The high degree of evolutionary conservation of the mTERF factor and its binding site [25] allows estimates of parameters *p* and *q* (the terminator passage probabilities in both directions) to be extrapolated among chordates. However, other parameters should be interpreted with caution. For example, the mouse possesses the terminator D-TERM, which is unknown in human and rat, between promoters LSP and HSP1 in the 5'-leader region of the tRNA-Phe gene [36]. Homologs of this terminator cannot be detected by sequence similarity even across close relatives (see Additional file 1, Section 5).

Our model’s predictions agree well with experimentally derived transcription levels in the mitochondria of frog, human and rat, including disease states such as the MELAS syndrome in human and the hypothyroid condition in rat.

The model predicts the intensities of polymerase-promoter binding, properties of the mTERF transcription terminator and absolute transcription levels of all mitochondrial genes (Figure 5 and Additional file 2). This is in contrast to the experiment which only provides relative measures of transcription of selected genes. An argument in favor of our model is the fact that all terminators are important in the prediction of transcription. Notably, excluding any of the terminators from modeling leads to predictions that intuitively are inadequate, e.g., transcription of only one DNA strand.

With a polymerase transcription rate of 500 nt/s, the model conforms better to the experiment (i.e. most predictions fall within experimental error) than with a rate of 200 nt/s. However, the rate choice needs further justification both *in silico* and in the experiment.

The concentration of the mtTFA transcription factor increases monotonically during early embryogenesis in frog [18]. mtTFA is a universal activator, and the expected increase of its binding intensities to all promoters is accurately predicted by the model (column LSP1, Table 4). There is no similar evidence for the mTERF factor, and the model does not predict the monotonic change either.

In mammals, the efficiency of the HSP1 promoter is predicted to be markedly greater than that of the HSP2 promoter on the heavy strand [5]. Notably, this result is not supported in our model, where *HSP2* is 4 times more efficient than HSP1.

In human, the predicted absolute transcription levels of protein-coding genes are unexpectedly low. One transcript is produced every 15–26 minutes, depending on the gene (Figure 5, Additional file 2), while the entire mitochondrial genome is transcribed in 33 s under the polymerase elongation rate of 500 nt/s. Such selective transcription agrees with previous measurements of absolute mRNA concentrations [37].

Furthermore, in human, transcription levels of protein-coding genes on the light and heavy strand are predicted to be very similar. This suggests that polymerases that initiate at promoters HSP1 and HSP2, and that pass the mTERF terminator collide very rarely. Such counterflows of polymerases are also rare for LSP-initiated polymerases that passed the first G-quadruplex terminator. There is negligible competition between free and bound polymerases for the promoter, because under high elongation rates and low binding intensities each promoter becomes available long before the next binding attempt. The probability that a promoter bound by a polymerase continues into the next circle is also low: in 9 hours of modeled time there are only 1 ± 1 such polymerases on the light strand and 23 ± 6 – on the heavy strand (average values ± unbiased standard deviation over *n* = 1000 trajectories). A possible biological explanation of such low polymerase competition in mitochondria of human is minimization of DNA damage during collisions. In plastids, the bacterial-type polymerases are considerably slower but collide frequently [1]. The bacterial-type polymerases inherited by mitochondria from their α-proteobacterial ancestors might have been lost and replaced by faster phage polymerases to reduce DNA mutation rate [38] (Part 3, Section 9.7).

In frog and rat, on the other hand, polymerase competition is more pronounced (see Figure 5 and Additional file 2), which can be explained by differences in RNA half-lives.

In human with MELAS syndrome, the model predicts a 1.21-fold decrease of the mTERF·DNA binding intensity and a 7.75-fold decrease of the HSP1 promoter efficiency. Transcription levels of tRNA-Phe and rRNA drop 3.84- and 1.2-fold, respectively, with possible implications for the MELAS phenotype. For example, consider the influence of rRNA. Highly expressed mRNAs are normally entirely covered by ribosomes that protect them from adverse modifications. The decreased expression of rRNA predicted by our model may lead to decreased ribosomal coverage of mRNAs and cause increased transcript damage (see Section 3 of the Methods). Similarly, we can consider the influence of tRNA-Phe: underexpression of tRNA-Phe attenuates translation and, at the same time, widens the window between ribosomes on RNA (see Section 3 of the Methods).

Increased transcription of CYTB relative to the upstream genes reported from the experiment cannot be predicted in the model. However, in thiamphenicol-treated cells the CYTB mRNA half-life shows a high degree of variation [39] (Table 2). This suggests a possible experimental bias in measuring the time and thus the CYTB transcription level. A slight discrepancy of 6% between the model and experiment for expression of the human ND2 gene might be explained by a similar bias in estimating half-lives [39] (Table 2).

In the model, intensities of the mTERF binding and LSP transcription initiation are equal in eu- and hypothyroid rats. In experiment, methylation of the mTERF binding site remains unaffected, and of the LSP promoter exhibits minimal changes [14], which agrees well with the stability of their predicted binding intensities.

Conversely, in the model, the total intensity *HSP* of transcription initiation from promoters HSP1 and HSP2 is 2.15-fold lower in the hypothyroid rat. In experiment, methylation of the HSP1 region changes considerably, but methylation of the HSP2 changes negligibly [14], which also agrees with the predicted decrease of transcription initiation from promoters HSP1 and HSP2.

## Conclusions

Our previous analyses [1] and current study demonstrate that the model produces results in good agreement with experimental evidence from plastids and mitochondria. It accurately predicts the RNA polymerase binding intensities, transcription terminator characteristics, and absolute transcription levels of all mitochondrial genes.

Individual gene transcription intervals are predicted to be long in human (15–26 min) and rat (2–10 min), but short in frog (8–25 sec). RNA polymerase competition is shown to be negligible in the mitochondria of human but evident in rat and frog, albeit much less intense compared to that in plastids. Advantages of the phage-type vs. bacterial-type RNA polymerases in mitochondria are suggested and discussed.

In hypothyroid rat, we describe how changes in methylation patterns of the mTERF binding site and three promoters correlate with intensities of the mTERF binding and transcription initiations.

We have also proposed a polysome-ribonuclease interaction model (see Section 3 of the Methods) and factors explaining the MELAS phenotype development in human.

## Competing interests

The authors declare no competing interests.

## Authors’ contributions

VAL, OAZ and AVS proposed the model, estimated its parameters, chose source data and participated in the analysis of results. VAL, SAP and AVS wrote Additional file 3 including derivation of formulas (5)–(7). LIR actualized the model implementation: wrote software, performed the computations and participated in the data preparation and analysis. OAZ performed the sequence alignment, the statistical analysis and participated in the data preparation and the computations. VAL, OAZ, AVS and LIR wrote the manuscript. All authors read and approved the final manuscript.

## Reviewers’ comments

*Reviewer’s report 1 – Dr. Anthony Almudevar*

The authors apply a parametric model for RNA polymerase interaction during transcription, developed in an earlier paper [1], which successfully predicted changes in gene transcription levels attributable to various experimental perturbations. The model is applied to new data, and appears to represent a significant extension of the model.

The reported predictions are quite interesting, but the paper as a whole seems very hastily written, often consisting of a sequence of brief paragraphs. This is especially true of Section [4. Modeling Procedure] on Page 13. The paper needs to be significantly reorganized.

**Response.** A sequence of brief paragraphs is justified by the intention to concisely list various experimental data and to state what the model is based on. Generally, the manuscript was reorganized (refer to response #2 to Reviewer 2), and currently this sequence is distributed between the main text and Supplement 1.

I think it would also be important to introduce a careful definition of the model, introducing notation for all parameters and observations at one place. The paragraph on page 8 beginning "The solution is a set of parameters ..." introduces λ, *p* and *q*, but more model elements are introduced at various subsequent places in the paper (including the Supplementary). It would better to see the full extent of the model, including a clear definition of the parameter space, at this point.

**Response.** The model definition in Section 1 of the Methods is extended and all model parameters are now grouped. Additionally, the software setup and execution parameters are briefly described.

Quality of written English: Needs some language corrections before being published.

**Response.** Done (see the last response to Reviewer 2).

*Reviewer’s report 2 – Prof. Marek Kimmel*

The model is an application of the previously published more general model of interaction of RNA polymerases, protein factors and secondary structure during transcription. Current version is limited to phage-type polymerases and transcription factors in the mitochondrial genomes of human, rat and frog.

Response #1. The current version of the model is more sophisticated: it now accounts for a protein-dependent terminator (mTERF) and completion of more than one circle on the mtDNA strand by RNA polymerases. Furthermore, the polymerase interaction described in [1] was not necessarily applicable to mitochondria, as the model behaves differently: it predicts high polymerase competition in plastids, and negligible competition in human mitochondria. Moreover, modeling the same mechanism in mitochondria provides explanations for the MELAS phenotype and the phenotype caused by hyposecretion of thyroid hormone. Although, different functional phenomena were explained in [1].

The model is based on comprehensive data and a detailed analysis of inferred parameter values. This is an interesting paper and it may be ultimately published. However, as it is now, it is overloaded with detail on one side and is missing some important information on the other. Accordingly, in my opinion, most of the details included in Sections 4-6 of the Background, should be moved to an online Supplement. Same is true of most of Section 4 of the Methods. What is to remain in the main body, are pointers to respective sections of the Supplement. Of Figures 1, 2 and 3, one might remain in the paper body. Background section is missing a description of the principles of the model. Instead, the authors seem to have decided that it will be enough to cite an earlier paper and defer the description (in a rudimentary form) to Section 5 of the Methods. This is unfortunate and I think should be corrected by expanding this material to a full-fledged description and moving it to the Background or to the beginning of the Methods. The part of Discussion in pp. 23-24 belongs more to the model description or to the Methods. After this "cleanup", the manuscript will be much more legible.

Response #2. Sections 4, 6 of the Background and section 4 of the Methods have been moved to Supplement 1 (Sections 1–3). Discussion in pp. 23-24 has been moved into the model description (Section 1 of the Methods). The model description in Section 1 of the Methods has now been extended and all model parameters grouped together.

Detailed remarks

Page 8. "Ann attempt is successful if the promoter is not occupied by a polymerase or factor." This seems unclear; the factors are supposed to attract polymerases (?)

**Response.** Transcription factors (proteins) binding DNA close to a promoter often act as activators or repressors of transcription. Such factors are common in plastids and mitochondria. In the mitochondria of metazoa and some protozoa, the mTERF factor has an important function as both a terminator (not bound to the promoter) and an activator. Modeling predicts the intensities of mTERF binding and transcription initiation attempts for all promoters that unlikely can be measured directly in the experiment.

Page 8, bottom. Define "contents".

**Response.** The "relative RNA content" is replaced with Â«relative RNA concentrationÂ»: *u*_*ij*_ is the RNA concentration of the *j-*th gene at *i*-th time point relative to the same concentration at the null time point, i.e., the ratio of two concentrations sampled during an experiment. Notably, the experiments do not allow measuring numerator and denominator of the ratio.

Page 9. What are *z*_*0j*_ and z_0_?

**Response.** Here *z*_*ij*_ is the transcription level of the *j*-th gene at the *i*-th time point, *z*_*0j*_ – transcription level of the same gene at 0-th time point, i.e., *u*_*ij*_ = *z*_*ij*_/*z*_*0j*_. Similarly, *z*_*j*_ is the transcription level of the *j*-th gene, *z*_*0*_ – the transcription level of the 0-th (reference) gene; we calculated *z*_*j*_/*z*_*0*_ to compare it with the experimentally determined *u*_*j*_.

Page 9. "hypothyroid or euthyroid". Rat ?

**Response.** Indeed, rat. Corrected.

Page 9. The "lower and upper parts of the ratio" are usually called numerator and denominator.

**Response.** Corrected.

Page 10. Define *x* and *y*.

**Response.** These variables *x*‾,*y*‾ have been eliminated.

Page 10. The list of "considered functionals" is superfluous.

**Response.** The list has now been moved to Supplement 1 (Section 4).

Page 11. The error analysis on this page is not very professional. A statistician should be consulted at least regarding notation and references.

**Response.** Following the reviewer's suggestion, we improved Section 4 of the Methods. According to Chapter 4, Section 4.3, of [28], “under the normal distribution of measurements and a large sample size approximately 70% of them belong within *x*‾±σ(*x*), where *x*‾ is the mean. Apparently, the standard deviation σ(*x*) bears the previously discussed meaning of “error”. If measurements of *x* are scarce, then their *error* is to be Δ*x* that equals σ(*x*)”.

From [28], equality Δ*x =* σ(*x*) is postulated also in cases of indefinite distributions of measurements. Rules of estimating the error of the sum, difference, product and ratio are given in Chapters 1, 2, 3 of [28], and are stated in Section 4 of the Methods. Tables 8-9 now contain errors estimated accordingly.

Page 14, top sentence. Is this stabilization a hypothesis, empirical observation, or conclusion bad on modeling?

**Response.** It is the result of the modeling.

Page 31. Should more details be included in the legend of Figure 4?

**Response.** Corrected.

Table 10. This Table has important information, but probably will be better off reworked into a bar chart. The table itself may be moved to the Supplement.

**Response.** Table 10 has now been moved to Supplement 3 and replaced by Fig. 5 in the main text.

Quality of written English: Not suitable for publication unless extensively edited

**Response.** The authors recruited native speakers to edit the language (mentioned in the Acknowledgements).

*Reviewer’s report 3 – Dr. Georgy Karev (nominated by Dr. Peter Olofsson)*

This work continues a recent paper of the same author's team [1], where a mathematical model of interaction and competition of many RNA polymerases transcribing simultaneously a DNA locus, was formulated and developed. The model was based on the concept of interactions suggested and developed by the authors. The concept has underlying "mechanistic image": similarly to "hard spheres", RNA polymerases are connected with DNA molecule and then move and come into collision with each other' according to certain stochastic rules. Surprisingly, a computer model based on this simplified mechanistic, not biochemical, description of interaction processes with more or less universal parameters was able to predict the expression levels of genes in different loci of plastids of plants in [1] and mitochondria of chordates (frogs, rats and human) in the present paper. Some other interesting phenotypic phenomena studied in both papers can also be explained within the frameworks of the developed model. Additionally, an interesting model of interaction between polysomes and ribonucleases was formulated in the present paper.

The algorithmic realization used a representation of the model as a multi-agent system; interactions between the agents were modeled using the Monte-Carlo method. The behaviors of agents at every instant were modeled by certain deterministic or stochastic processes, and corresponding events happened randomly and asynchronously. The model uses non-uniform discrete time moments, which correspond to the moments of event occurrences.

Each run of the program models a single realization of inter-cell processes. Biological experiments use tissues composed from many millions of cells implying a result naturally averaged over all cells. Similar result form *in silico* experiments can be obtained only by multiple run of the program and subsequent averaging of the results. It demands a powerful computer system and a long time for computations.

Overall, the work presents a complex model based on non-trivial biological background and mathematical theory of stochastic processes, which was

realized as a program package for multiprocessor computer; the authors used a cluster with 2048 processors and nevertheless the computations took a long time. The model has explanatory properties and also allows predicting the transcription levels of future experiments. The program package, the manual and tutorial examples are available on the laboratory site. It would be very helpful for readers if the manual would be preceded by non-formal but complete enough description of the model.

Suggested changes:

The authors refer to their previous paper [1] for the model description, so it would be helpful to give a summary of the model (background, main assumptions, variables and processes).

**Response.** See response #2 to reviewer 2.

A logical path from biological motivation and "verbal" model to computer model is not entirely clear from the text. In particular, mathematical scheme of the model and especially the computer algorithm contain some additional assumptions about interactions between deterministic and stochastic processes that are accounted for the model; these assumptions were realized in the program but they do not follow immediately from the biological background. Supplementary materials contains enough explanations for general understanding of the model but it would be helpful to extend it or to give additional explanations for missing mathematical details.

**Response.** Each point in the description of both models (Sections 1 and 3 of the Methods) contains assumptions, including the Poisson nature of the flows, discrete time, averaging over trajectories with the Monte-Carlo approach, ordering the queue, modeling parameter settings (such as the 9 hrs time period to count transcriptions), etc.

A non-trivial proof of the auxiliary model (Supplement 2) is contained in equation (*): the probability of RNA cleavage inside the spliceosome during short time Δ*t* in a window equals the product of *μ*Δ*t* + *o*(Δ*t*) (the probability of RNA cleavage in the window) and $\frac{\exp\left(-\lambda w\right)}{1+d\lambda }$ (the probability of existence of this window during Δ*t*). The difficulty here is the formal assumption of a stationary process (lack of cleavage) to describe the probability of opening the window for a generally non-stationary process. A rigorous mathematic study of this non-stationary process is sophisticated and is not discussed in this work.

The model is a complex and rather specific system of interconnected stochastic processes. Computer realization of complex systems and computations even of a large number of individual trajectories cannot guarantee that some important qualitative peculiarities of the system behavior were not missed. Are any general mathematical results about qualitative behavior of the model known by the authors?

**Response.** This is an important and interesting point. Unfortunately, many particularly mathematical questions remain unanswered in this model, e.g., an explicit description of the stochastic process of effective (in terms of the “intensity”) polymerase-promoter binding attempts even under a fixed arrangement of only two opposite promoters.

A large number of deterministic or stochastic events that happen in random time moments were taken into consideration in the model. Computer realization of any flow of events demands some kind of ordering of the events, because they are considered "one by one" in a computer. The way of ordering of the event flow generally imposes some additional restrictions and conditions in the initial model. The authors give only a few explanations of how the ordering of event flow is organized in the computer model.

**Response.** Despite the fact that all processes in the model initiate at the same “zero” time, the first polymerase binds to each promoter after a random time determined by the Poisson process associated with that promoter. It is unlikely that these precisely calculated times coincide for different promoters, therefore subsequent discrete “moves” of all polymerases also occur at different time-points, because they have the same rate. Thus we may assume simultaneous events do not occur; in particular, any collision always involves one moving object and one stationary object. This is the sole assumption and it is physically plausible. This assumption allows us to use non-uniform discrete time instead of continuous time. Once a future event is determined to occur at some time, it is inserted in the priority queue according to that time. Each step of the modeled time involves extraction of the next event from the queue top. The priority queue must be capable of containing tens of thousands of time-ordered events which causes serious algorithmic and computational difficulties. The program performance depends heavily on the speed of the queue servicing. Currently the queue is implemented as a binary heap, and the average performance we reached in the model is one to two orders of magnitude faster than modeled physical time.

The authors describe in good details the biological background of the transcription process, which the model was based on. It would be helpful to point out those phenomena and processes, which were not accounted in the model. It would help to understand better the boundaries of applicability of the model and possible ways to its improvement.

**Response.** A number of transcription-related processes are not considered in this study. For example, the rate of the phage type polymerase is likely to be affected by RNA primary and secondary structures. Transcription initiation factors mentioned in the Background are not considered here. Termination at a G-quadruplex is not modeled. Only the experimental ratio of terminating polymerases is considered. The formation of a supercoil and Z-DNA in front of the moving polymerase can lead to its remote interaction with other protein factors.

The authors pointed out in their previous paper that nucleotide composition of DNA was not accounted for in the existing version of the program (more exactly, it was used only for other objects to be related with certain position of the DNA sequence), and that the nucleotide composition of DNA can be taken into account in the next versions of the program. By now having more experience with the program, do the authors believe that more explicit account of nucleotide composition is necessary or desirable, and if so, what are the corresponding problems?

**Response.** The authors developed a model and its computer realization that describe the effect of the RNA primary and secondary structures on the rate and nature (pause, arrest, termination) of the polymerase movement [40]. However, incorporating these would lead to reduced performance of the software in its current implementation.

The authors have concluded that the half-life of mRNA in human mitochondria can be changed significantly even after small variations of transcription intensity; for this reason it is not clear why they take equal half-life values for mitochondria of a healthy human and a human with MELAS syndrome.

**Response.** This prediction is qualitative. At least, both our models predict slight changes in transcription of selected genes associated with the MELAS phenotype.

Two models are described in this study: the main model of RNA-polymerase interaction, and the auxiliary model of mRNA-ribonuclease interaction in the polysome. The main model predicts gene transcription levels, the auxiliary model specifies RNA half-lives. The two models can be pipelined iteratively, but this was not included in the design of this study.

The authors gave the results at the speed of RNA polymerase equal to 200 and 500 nt/s. Why were these values chosen? Do there exist similar results with other speed values, e.g., 800 or 1200 nt/s?

**Response.** Predictions under rates of 800 and 1200 are not provided here. Our tests suggest that the rate of 800 produces even closer agreement with the experiment compared to the rate of 500, whereas under 1200 this congruence is poor. A preliminary prediction of the model is that the polymerase elongation rate is 500–800 nt/s.

Is it possible to obtain similar results for other species, e.g., for chicken and yeast?

**Response.** The yeast mitochondrial genome is known to contain 19 promoters. Solving an inverse problem is very computationally consuming under so many unknown parameters, which exceeds available hardware resources. In chicken, mitochondrial transcription is initiated at a single bidirectional promoter or two overlapping promoters. However, more experimental evidence on gene expression is needed. Therefore, predictions are not provided for these organisms.

Quality of written English: **Acceptable.**

## Supplementary Material

Additional file 1 **Supplement 1. RNA half-lives, mTERF-independent transcription termination, modeling procedure.** Click here for file

Additional file 2 **Supplement 2. Transcriptions predicted during 9 hours of modeled physical time.** Click here for file

Additional file 3 **Supplement 3.  Details of the auxiliary model.** Click here for file
