# Supplementary material for: Modeling RNA polymerase interaction in mitochondria of chordates
Source: Biol Direct. 2012 Aug 9;7:26. doi: 10.1186/1745-6150-7-26 (PMC3583402; doi:10.1186/1745-6150-7-26)
Supplement: Additional file 1 — Supplement 1. RNA half-lives, mTERF-independent transcription termination, modeling procedure. [file 1745-6150-7-26-S1.doc]

**Supplement 1**

**1**. **RNA half-lives**

RNA stability in human mitochondria was studied in [37] and [39][[1]](#footnote-2). The half-lives, in minutes, of heavy-strand transcripts in mitochondria of healthy human (mean ± standard deviation) are: 219 ± 22 for ND1; 142 ± 3 for ND2; 204 ± 91 for COX1; 297 ± 97 for COX2; 424 ± 104 for ATP6/8; 59 ± 1 for ND3; 120 ± 27 for ND5; 132 ± 24 for CYTB. The half-life of rRNA is of order of several hours (Table 8) [[2]](#footnote-3).

## Table 8 - Experimental data on mitochondrial transcripts in healthy human

Relative mRNA concentrations in stable state as percent of ND1 mRNA concentration: the ± signs stand for confidence levels. For half-lives standard deviations are provided. Data are from [39] and [37].

| Gene | Untreated cells | | Thiamphenicol treated cells | | Gene |
| --- | --- | --- | --- | --- | --- |
|  | Steady-state level | Half-life (min) | Half-life (min) | Relative change | length |
| 16S |  | 180 ± 30 |  |  | 1558 |
| ND1 | 100 ± 4 | 219 ± 22 | 273 ± 21 | 1.25 | 956 |
| ND2 | 91 ± 11 | 142 ± 3 | 296 ± 22 | 2.09 | 1042 |
| COX1 | 97 ± 19 | 204 ± 91 | 236 ± 65 | 1.15 | 1542 |
| COX2 | 234 ± 19 | 297 ± 97 | 277 ± 78 | 0.94 | 684 |
| ATP6/8 | 177 ± 69 | 424 ± 104 | 506 ± 51 | 1.19 | 842 |
| ND3 | 28 ± 1 | 59 ± 1 | 132 ± 16 | 2.23 | 346 |
| ND5 | 102 ± 17 | 120 ± 27 |  |  | 1812 |
| CYTB | 139 ± 16 | 132 ± 24 | 406 ± 27 | 3.06 | 1141 |

RNA half-lives in isolated mitochondria in rat were measured for the normal level of thyroid hormone (euthyroid individuals) and under hormone deficiency (hypothyroid rats) [14]. In euthyroid rat, the half-lives in minutes (mean ± standard deviation) are: 44.48 ± 6.34 for 16S RNA, 46.00 ± 10.41 for ND5, 84.41 ± 27.49 for ND4/4L & COX1, 63.70 ± 7.82 for CYTB, 78.14 ± 21.05 for ATP6/8 & COX3. These half-lives are substantially shorter than in human (Table 6). In hypothyroid rats, the half-lives are 2.13-fold greater, on average.

Although RNA half-lives are not known for frog, this does not preclude comparisons between the model predictions and experimental data on relative gene expression levels that do not depend on RNA decay times.

**2**. **mTERF-independent transcription termination**

In human mitochondria the mTERF-independent terminator occupies positions 282–300 on the light strand, terminating about 65% of transcripts initiated at the LSP promoter [41]. This terminator is strictly polarized, because termination requires the formation of a G-quadruplex in RNA as well as a downstream stretch of poly-dT DNA. In human, the terminator is a sequence of 12 guanine nucleotides interrupted by one internal adenine. Termination occurs when the G-quadruplex is formed in proximity to the RNA polymerase.

Non-protein terminators are common for all phage-type RNA polymerases. Putative terminator regions in three model species are shown in Table 9. In this work, these terminator regions have been predicted *in silico* in rat and frog. Termination is likely to occur 10-15 nucleotides downstream of these regions, as observed in human. The G-rich region is known to be a long-transcript processing (cleavage) site in frog [42].

## Table 9 - Factor-independent transcription terminators: G-rich sites in mitochondria

Positions in the complement L-strand are shown in parentheses.

| Species | Sequence | Location | Composition |
| --- | --- | --- | --- |
| *Homo sapiens* | [Genbank:NC_012920.1] | (16086..16098) | GGGGGAGGGGGGG |
| *Rattus norvegicus* | [Genbank:NC_001665.2] | (303..315) | GGGGGTGGGGGGG |
| *Xenopus laevis* | [Genbank:NC_001573.1] | (1808..1819) | GGGGGGTAGGGGG |

**3**. **Modeling procedure**

Experimental estimates of relative RNA concentrations in frog were taken from [19]. For healthy human, estimates of relative rRNA concentrations were taken from [37], and refined estimates of mRNA from Table 2 in [39]. Relative RNA concentrations for human with MELAS syndrome were taken from Table 1 in [26], and for both rats – from Fig. 1 in [14].

These data were discussed in the Background section of this work and shown in Tables 4, 6, and 8.

All gene transcription levels stabilize in the model after less than 9 hours of modeled physical time. This time exceeds the half-lives of all rRNAs [37] (Table 1) and mRNAs [39] (Table 4) in the mitochondria of healthy human liver and rats [14] (Table 5), as well as exceeding the cell cycle period in frog embryos [43].

For all model organisms the following *general conditions* were imposed on the solution:

1) Gene transcription levels take non-zero values. Moreover, each gene produces at least two RNAs during the period of an RNA half-life, when this half-life is known.

2) All parameters take positive values; *p*, *q* vary between 0 and 1, and $q\leq p$, to conform with the fact that passage on the heavy strand has been shown experimentally to be markedly greater than passage on the light strand, i.e., $0<q\leq p<1$. Intensity parameters *LSP* and *HSP* vary between 0.002 and 0.1 (s ^-1^), intensity *mTERF* – between 0.002 and 1 (s ^-1^). Otherwise gene transcription levels do not stabilize in the model even after the model run-time considerably exceeded 9 hours. Note that gene transcription levels do not increase under *mTERF* > 1, as here higher attempt intensities do not lead to higher attempt success.

In frog, the functional *L*_1_*n*(total) from (4) was used. Its global minimum (the solution) defined precise values of the parameters *p* and *q*. In addition, the parameters of intensities *mTERF* and *LSP1* of binding to the mTERF site and LSP1 promoter, respectively, were found at each of 10 (first frog), 7 (second frog) and 6 (third frog) time points. Thus, *L*_1_*n*(total) was a functional of 48 variables. The solutions are given in Tables 3, 4.

Since the mTERF terminator and its binding site on mtDNA are highly conserved [25], estimates of *p* and *q* obtained for frog were used for human (healthy and diseased) and rat (euthyroid and hypothyroid).

The functional *L*_1_*n* from (3) was used in healthy human. Its global minimum defined the intensities of binding attempts of RNA polymerases to promoters LSP, HSP1, HSP2, and of the protein terminator mTERF to its site.

The following *additional conditions* were imposed on the solution in the human case:

3) When comparing healthy and diseased humans, the difference in transcription levels of the tRNA-Leu(UUR) and tRNA-Lys genes does not exceed 20% and 50%, respectively [26, Table 1].

4) *LSP* = *LSP–* and *HSP2* = *HSP2–*  (where *LSP–* and *HSP2–* are the intensities of binding with promoters LSP and HSP2 in the presence of the MELAS mutation), because the mutation has no pronounced effect on transcription initiation from these promoters. Its impact is known for promoter HSP1 [11].

5) *mTERF* > *mTERF–* and *HSP1* > *HSP1–*  (where *mTERF–* and *HSP1–*

are defined analogously to the above), since the MELAS mutation effectively lowers the mTERF affinity for both sites during its cooperative binding with HSP1. Indeed, the absolute bond energy of the mTERF∙DNA complex continuously increases monotonically with the complex’s half-life, which decreases 7–10 fold with the MELAS mutation. The energy also monotonically increases with the increase of terminator binding intensity [26] (Fig. 5 and its discussion). However, the form of these dependencies is not clear.

6) In humans, 1.16< RNA/RNA– <1.22. In the fraction, RNA (or RNA_) is the sum of values $2 l\cdot t\cdot z$ (gene length multiplied by its RNA half-life, Table 8, and its transcription level) over all genes in healthy (or diseased) human. We used experimentally obtained ratios of total RNA to total DNA [26] (Table 1) to estimate the lower and higher bounds in condition 6, because the amounts of DNA are canceled. RNA half-lives were assumed equal in healthy and diseased human, see the Discussion.

7) The *ratio R* of gene 12S to gene COX2 transcription levels exceeds 16.9. This is in accordance with the published transcription levels for these 2 genes [37], 12S and COX2 RNA concentrations are 12600 and 225 molecules per cell (Table 2 in [37]), while upper and lower bounds for their half-lives are 146 and 44 minutes (Table 1, experiment 4 in [37]), which gives the lower estimate of *R* > 17. Similarly, the higher estimate of *R* < 27 can be obtained from [37] (Table 1, experiment 3). The COX2 gene was chosen as a reference because its RNA half-life is robust to various perturbations, including antibiotic treatments [39] (Table 4). We also found the following *R* values for other genes: 19 < *R* < 37 for ATP6/8, 17 < *R* < 27 for COX2, 20 < *R* < 25 for COX3, 17 < *R* < 25 for CYTB, 41 < *R* < 52 for COX1. Therefore, we assume 17 < *R* < 37. The special case of COX1 may suggest an experimental bias, as all the above genes are located on the same DNA strand lacking promoters and terminators and thus are expected to follow similar expression patterns.

The functional *L*_1_*n* was minimized over eight variables and under the above described conditions. The solutions are given in Table 5.

In rat, the same functional was minimized over six variables, the parameters of *LSP*, *HSP* = *HSP1*+*HSP2*, *mTERF*. The following *additional* conditions were imposed.

8) *LSP* = *LSP–* and *HSP2* = *HSP2–* (where *mTERF–* and *HSP1–* are as above), which reflects a small change in methylation of the corresponding promoters [14];

9) In euthyroid rat, 17 < *R* < 60. The lower bound is assumed equal to that in healthy human, the higher bound is assumed equal to that in mouse [36].

**4**. **The list of additionally analyzed functionals** are the following:

$$L_{2}n=\sqrt{\sum_{ji} \left(\frac{x_{ji}-y_{ji}}{\max\{x_{ji},y_{ji}\}}\right)^{2}}; L_{1}=\sum_{ji} \left|x_{ji}-y_{ji}\right| ; L_{2}=\sqrt{\sum_{ji} \left(x_{ji}-y_{ji}\right)^{2}};$$

;

$\hat{\chi}^{2}=\sum_{ji} \frac{\left(a_{ji}-b_{ji}\right)^{2}}{a_{ji}}\text{ , where }a_{ji}=\frac{x_{ji}}{\sum_{ji} x_{ji}}, b_{ji}=\frac{y_{ji}}{\sum_{ji} y_{ji}}$;

$\chi^{2}=\sum_{ji} \frac{\eta_{ji}\left(a_{ji}-b_{ji}\right)^{2}}{b_{ji}}\text{ , where }\eta_{ji}=\exp\left(-\Delta x_{ji}/x_{ji}\right)$;

$\hat{S}=\sqrt{\sum_{ji} \left(\frac{x_{ji}}{a}+\frac{y_{ji}}{b}\right)^{2}}\text{, where }a=\sum_{ji} x_{ji}, b=\sum_{ji} y_{ji}$;

$S=\sqrt{\sum_{ji} \left(\frac{x_{ji}}{a}+\frac{y_{ji}}{b}\right)^{2}}\text{ , where }a=\sum_{ji} \frac{x_{ji}}{\eta_{ji}}, b=\sum_{ji} \frac{y_{ji}}{\eta_{ji}}, \eta_{ji}=\exp\left(-\Delta x_{ij}/x_{ij}\right)$.

## 5. 5'-Leader regions of tRNA-Phe gene

The D-TERM terminator sequence is underlined. 3'-coordinates are given for leader regions (coordinates are “–1” relative to the gene start). Sequences do not align.

| *Mus musculus* | ACCAAAACTCTAATCATACTCTATTACGCAATAAACATTAACAA | 16299 |
| --- | --- | --- |
| *Rattus norvegicus* | GCCTACCCT---CAGAAAATTCCACATACACCAAA--------- | 16313 |
| *Homo sapiens* | GCTAACCCCATACCCCGAACCAACCAAACCCCAAAGACA----- | 577 |

Additional references

41. Wanrooij PH, Uhler JP, Simonsson T, Falkenberg M, Gustafsson CM: **G-quadruplex structures in RNA stimulate mitochondrial transcription termination and primer formation.** *Proc Nat Acad Sci USA* 2010, **107:**16072–16077.

42. Bogenhagen DF, Morvillo MV: **Mapping light strand transcripts near the origin of replication of** ***Xenopus laevis*; mitochondrial DNA.** *Nucleic Acids Res* 1990, **18:**6377–6383.

43. Cooper GM: *The Cell: A Molecular Approach*. 2nd edition. Sunderland: Sinauer Associates; 2000.

1. Most citations in this Supplement refer to the list in the main body text. Few extra references are provided in the end of this file. [↑](#footnote-ref-2)
2. Tables in this Supplement are numbered to continue the sequence of tables in the main body text, which are also cited in this file. [↑](#footnote-ref-3)
