# Supplementary material for: Modeling RNA polymerase interaction in mitochondria of chordates
Source: Biol Direct. 2012 Aug 9;7:26. doi: 10.1186/1745-6150-7-26 (PMC3583402; doi:10.1186/1745-6150-7-26)
Supplement: Additional file 2 — Supplement 2. Transcriptions predicted during 9 hours of modeled physical time. [file 1745-6150-7-26-S2.doc]

## Supplement 2: Transcriptions predicted during 9 hours of modeled physical time

Average values and standard deviations are provided for 1000 model realizations; predictions for frogs are at +48h postfertilization time. “Competition” provides the percentage of polymerases terminated upon collision within regions confined between the mTERF site and tRNA-Thr gene (on heavy strand), and between tRNA-Pro and tRNA‑Gln genes (on light strand).

|  | *Xenopus laevis* | | | *Homo sapiens* | | *Rattus norvegicus* | |
| --- | --- | --- | --- | --- | --- | --- | --- |
| Gene | Frog 1 | Frog 2 | Frog 3 | WT | MELAS | Euthyroid | Hypothyroid |
| **H-strand:** |  |  |  |  |  |  |  |
| tRNA-Phe | 3074 ± 271 | 5003 ± 818 | 2835 ± 1345 | 123 ± 27 | 32 ± 24 | 1215 ± 51 | 556 ± 37 |
| 12S | 3004 ± 276 | 4931 ± 831 | 2815 ± 1348 | 527 ± 24 | 438 ± 21 | 2362 ± 46 | 1090 ± 34 |
| tRNA-Val | 2998 ± 277 | 4926 ± 830 | 2813 ± 1348 | 527 ± 24 | 438 ± 21 | 2361 ± 46 | 1090 ± 34 |
| 16S | 2865 ± 288 | 4799 ± 855 | 2779 ± 1351 | 527 ± 24 | 438 ± 21 | 2323 ± 46 | 1073 ± 35 |
| tRNA-Leu | 2012 ± 272 | 3981 ± 808 | 2495 ± 1346 | 22 ± 5 | 19 ± 5 | 257 ± 19 | 86 ± 9 |
| ND1 | 1934 ± 274 | 3908 ± 819 | 2475 ± 1349 | 22 ± 5 | 19 ± 5 | 229 ± 19 | 75 ± 9 |
| tRNA-Ile | 1929 ± 275 | 3903 ± 820 | 2474 ± 1349 | 22 ± 5 | 19 ± 5 | 227 ± 19 | 74 ± 8 |
| tRNA-Met | 1920 ± 276 | 3895 ± 820 | 2471 ± 1349 | 22 ± 5 | 19 ± 5 | 223 ± 19 | 72 ± 8 |
| ND2 | 1849 ± 283 | 3828 ± 833 | 2454 ± 1349 | 22 ± 5 | 19 ± 5 | 197 ± 18 | 62 ± 8 |
| tRNA-Trp | 1844 ± 282 | 3824 ± 834 | 2453 ± 1350 | 22 ± 5 | 19 ± 5 | 195 ± 18 | 62 ± 8 |
| COX1 | 1745 ± 284 | 3720 ± 856 | 2426 ± 1352 | 22 ± 5 | 18 ± 5 | 156 ± 16 | 48 ± 7 |
| tRNA-Asp | 1737 ± 284 | 3713 ± 857 | 2424 ± 1352 | 22 ± 5 | 18 ± 5 | 153 ± 16 | 47 ± 7 |
| COX2 | 1703 ± 285 | 3676 ± 861 | 2416 ± 1352 | 22 ± 5 | 18 ± 5 | 141 ± 15 | 42 ± 7 |
| tRNA-Lys | 1700 ± 286 | 3673 ± 861 | 2415 ± 1353 | 22 ± 5 | 18 ± 5 | 140 ± 15 | 42 ± 7 |
| ATP6/8 | 1665 ± 289 | 3638 ± 866 | 2405 ± 1353 | 21 ± 5 | 18 ± 5 | 125 ± 15 | 37 ± 7 |
| COX3 | 1634 ± 290 | 3604 ± 868 | 2397 ± 1354 | 21 ± 5 | 18 ± 5 | 114 ± 14 | 34 ± 6 |
| tRNA-Gly | 1631 ± 290 | 3601 ± 868 | 2396 ± 1354 | 21 ± 5 | 18 ± 5 | 113 ± 14 | 33 ± 6 |
| ND3 | 1614 ± 293 | 3584 ± 869 | 2391 ± 1355 | 21 ± 5 | 18 ± 5 | 109 ± 14 | 32 ± 6 |
| tRNA-Arg | 1611 ± 293 | 3582 ± 869 | 2390 ± 1354 | 21 ± 5 | 18 ± 5 | 108 ± 14 | 31 ± 6 |
| ND4 | 1524 ± 291 | 3487 ± 878 | 2364 ± 1357 | 21 ± 5 | 18 ± 5 | 87 ± 12 | 25 ± 5 |
| tRNA-His | 1520 ± 290 | 3481 ± 879 | 2363 ± 1357 | 21 ± 5 | 18 ± 5 | 86 ± 12 | 25 ± 5 |
| tRNA-Ser2 | 1516 ± 291 | 3477 ± 880 | 2362 ± 1357 | 21 ± 5 | 18 ± 5 | 86 ± 13 | 24 ± 5 |
| tRNA-Leu2 | 1510 ± 291 | 3472 ± 879 | 2361 ± 1358 | 21 ± 5 | 18 ± 5 | 85 ± 13 | 24 ± 5 |
| ND5 | 1400 ± 288 | 3323 ± 892 | 2319 ± 1365 | 21 ± 5 | 18 ± 5 | 67 ± 10 | 19 ± 5 |
| CYTB | 1273 ± 282 | 3120 ± 886 | 2259 ± 1369 | 21 ± 5 | 18 ± 5 | 53 ± 10 | 15 ± 4 |
| tRNA-Thr | 1267 ± 281 | 3111 ± 887 | 2255 ± 1369 | 21 ± 5 | 18 ± 5 | 53 ± 10 | 15 ± 4 |
| Competition | 37% | 22% | 10% | 5% | 5% | 79% | 83% |
| **L-strand**: |  |  |  |  |  |  |  |
| tRNA-Pro | 1605 ± 143 | 1585 ± 221 | 741 ± 151 | 35 ± 7 | 36 ± 5 | 1236 ± 34 | 1248 ± 40 |
| tRNA-Glu | 1505 ± 150 | 1421 ± 229 | 693 ± 152 | 35 ± 7 | 36 ± 5 | 1227 ± 34 | 1245 ± 40 |
| ND6 | 1469 ± 153 | 1368 ± 233 | 677 ± 151 | 35 ± 7 | 36 ± 5 | 1222 ± 34 | 1244 ± 40 |
| tRNA-Ser | 1124 ± 150 | 972 ± 214 | 572 ± 144 | 34 ± 7 | 35 ± 5 | 1133 ± 36 | 1215 ± 40 |
| tRNA-Tyr | 1040 ± 151 | 883 ± 198 | 549 ± 144 | 34 ± 7 | 35 ± 5 | 1099 ± 37 | 1203 ± 41 |
| tRNA-Cys | 1036 ± 149 | 879 ± 196 | 548 ± 144 | 34 ± 7 | 35 ± 5 | 1098 ± 37 | 1202 ± 41 |
| tRNA-Asn | 1030 ± 149 | 871 ± 194 | 547 ± 144 | 34 ± 7 | 35 ± 5 | 1095 ± 37 | 1202 ± 41 |
| tRNA-Ala | 1026 ± 151 | 867 ± 194 | 546 ± 143 | 34 ± 7 | 35 ± 5 | 1094 ± 37 | 1201 ± 41 |
| tRNA-Gln | 941 ± 147 | 788 ± 184 | 525 ± 140 | 34 ± 7 | 35 ± 5 | 1062 ± 37 | 1188 ± 40 |
| Competition | 41% | 50% | 29% | 3% | 3% | 14% | 5% |
