# Supplementary material for: Modeling RNA polymerase interaction in mitochondria of chordates
Source: Biol Direct. 2012 Aug 9;7:26. doi: 10.1186/1745-6150-7-26 (PMC3583402; doi:10.1186/1745-6150-7-26)
Supplement: Additional file 3 — Supplement 3. Details of the auxiliary model. [file 1745-6150-7-26-S3.doc]

**Supplement 3**

Consider the ribosome elongation rate $V$= 15 codons per second. The ribonuclease is assumed to bind with a *single specific* mRNA site of length *Vw* only if it is entirely free of any ribosome. The ribonuclease binding attempt is described by a Poisson flow with intensity $\mu$; a successful binding results instantly in mRNA cleavage. At translation initiation, the ribosome covers an mRNA region of length *Vd* that includes the start codon. This region is assumed to be 5'-terminal. Translation initiation attempts are described by a Poisson flow with intensity $\lambda$. As before, an attempt is successful if and only if the binding sites of the ribonuclease (RNBS) or ribosome (RBS) are not occupied by other ribosomes.

Consider that a window is a region of RNA between two neighboring ribosomes with a minimum length *Vw*. If the window contains an RNBS it can bind a ribonuclease.

Initiations are separated by time *d* when no initiation events are allowed. Average amount of initiation attempts in time *T* is $T\lambda$ and is the sum of average amount *Y* of successful initiations and average amount $Yd\lambda$ of failed attempts.

Then $Y=\frac{T\lambda}{1+d\lambda}$.

The probability of window formation in one mRNA is $\exp(-\lambda w)$. Then the average amount of windows formed in time $T$ in one mRNA is $X=Y\exp(-\lambda w)=$ $\frac{T\lambda\exp(-\lambda w)}{1+d\lambda}$.

Define *residual time* as a period between successive binding of two ribosomes excluding time *w* of forming a new window. Average residual time in one window on one mRNA is $1/\lambda$. Define *T* as an arbitrary time period. Average total residual time over all windows formed on one mRNA in time *T* is $X/\lambda=\frac{T\cdot\exp(-\lambda w)}{1+d\lambda}$. All windows pass the RNBS in a time period that also equals *T*. Thus, any mRNA is available to a ribonuclease during$X/\lambda T$of time *T*, i.e. the probability of it being available at any time point is $\frac{\exp(-\lambda w)}{1+d\lambda}$.

The probability of RNA cleavage in time $\Delta t$ is

$\mu\Delta t\cdot\frac{\exp(-\lambda w)}{1+d\lambda}+o(\Delta t)$.

This equation accounts for the RNBS being occupied over some period within $\Delta t$.

Therefore, in $\frac{dM}{dt}=-\kappa M$, where *M* is the number of polysomes, coefficient is $\kappa=\frac{\mu\cdot\exp(-\lambda w)}{1+d\lambda}$. Note that without accounting for ribonucleases the described process is stationary, and is nonstationary otherwise. Accordingly, the amount of polysomes exponentially decreases with time.

Then, the average RNA life time is $\frac{1}{\kappa}=\frac{1}{\mu}(1+d\lambda)\exp(w\lambda)$, and its half-life is $\tau=\frac{1}{\mu}(1+d\lambda)\exp(w\lambda)\ln2$.

Each mRNA was assumed above to possess a single ribonuclease site. The authors are unaware of the actual number and arrangement of cleavage sites on RNA. If their average number per a plastid RNA is *k*, then in the above equation $\kappa$ is substituted by $\kappa k$. Indeed, the amount of RNAs decaying in small time *dt* is proportional to the total amount of cleavage sites available in that time.
